# Elevated Midtrimester Triglycerides as a Biomarker for Postpartum Hyperglycemia in Gestational Diabetes

**DOI:** 10.1155/2020/3950652

**Published:** 2020-04-23

**Authors:** Mengyu Lai, Fang Fang, Yuhang Ma, Jiaying Yang, Jingjing Huang, Na Li, Mei Kang, Xianming Xu, Jiarong Zhang, Yufan Wang, Yongde Peng

**Affiliations:** ^1^Department of Endocrinology and Metabolism, Shanghai General Hospital, Shanghai Jiao Tong University, Shanghai, China; ^2^Clinical Research Center, Shanghai General Hospital, Shanghai Jiao Tong University, Shanghai, China; ^3^Department of Obstetrics and Gynecology, Shanghai General Hospital, Shanghai Jiao Tong University, Shanghai, China

## Abstract

**Background:**

Whether elevated triglyceride (TG) levels during pregnancy were a biomarker for postpartum abnormal glucose metabolism (AGM) in women with previous gestational diabetes mellitus (GDM) remained unknown. The aim of this study was to investigate the association between TG levels during the second trimester and postpartum AGM in GDM women.

**Methods:**

This was a retrospective cohort study including 513 GDM women. A 75 g oral glucose tolerance test (OGTT) was performed, and lipid levels were determined during pregnancy and the postpartum period. GDM patients were categorized into tertiles according to their TG levels at 24–28 weeks of gestation (TG < 2.14 mmol/L, TG: 2.14–2.89 mmol/L, and TG > 2.89 mmol/L). A logistic regression model was used to calculate the odds ratios (ORs) and 95% confidence intervals (CIs).

**Results:**

During pregnancy, women in the high TG tertile showed higher HbA1c levels (5.47 ± 0.58% versus 5.28 ± 0.49%, *p* = 0.006), higher total cholesterol (TC) levels (5.85 ± 1.23 mmol/L versus 5.15 ± 0.97 mmol/L, *p* = 0.026), and higher HOMA-IR (2.36 (1.62-3.45) versus 1.49 (0.97-2.33), *p* < 0.001) than the participants in the low TG tertile. After delivery, the prevalence rates of AGM based on above tertiles of TG levels during pregnancy were 26.90%, 33.33%, and 43.27%, respectively (*p* = 0.006). High TG tertile during the second trimester was associated with the presence of postpartum AGM (adjusted OR: 2.001, 95% CI: 1.054-3.800, *p* = 0.034).

**Conclusions:**

The elevated midtrimester TG levels were not only accompanied by higher glucose and lipid levels and more severe insulin resistance at the time of the measurement but were a biomarker for postpartum AGM as well.

## 1. Introduction

Gestational diabetes mellitus (GDM) is a pregnancy-related metabolic disorder, which affects 4–18% of pregnant women in various countries [1]. GDM not only increases the risk of maternal and fetal perinatal complications in the short term but also increases the risk of subsequently developing type 2 diabetes mellitus (T2DM) and cardiovascular disease in the long term [2–5]. The American Diabetes Association (ADA) recommends performing a 75 g oral glucose tolerance test (OGTT) over hemoglobin A1c (HbA1c) at 4-12 weeks postpartum for every GDM patient to evaluate her glucose metabolism [6, 7]. Intensified surveillance, early detection of risk factors, and active intervention may reduce postpartum abnormal glucose metabolism (AGM).

An elevated triglyceride (TG) profile is a common lipid abnormality that accompanies T2DM and prediabetic states [8]. Hyperlipidemia is more severe in pregnant women with GDM than in normal glucose pregnant women [9]. Studies indicated that maternal lipid, which changed throughout gestation to meet the needs of fetal growth, played an important role on obstetric complications [10]. Further, prospective studies showed that the TG level was an independent risk factor for developing diabetes in middle-aged women [11, 12]. In a prospective case-control study, higher TG levels were observed at 11-14 weeks of gestation in women with GDM, and multivariate regression analysis indicated that TG levels were a significant predictor of a subsequent diagnosis of GDM [13]. However, fewer studies have characterized the relationship between TG levels during pregnancy and the risk of postpartum AGM in GDM patients.

Here, we explored the relationship between TG levels during midterm pregnancy and postpartum glucose metabolism in Chinese women with previous GDM. We aimed to determine whether midterm elevated TG levels were a biomarker for the development of sustained hyperglycemia after delivery.

## 2. Materials and Methods

### 2.1. Subjects

**Figure 1:**
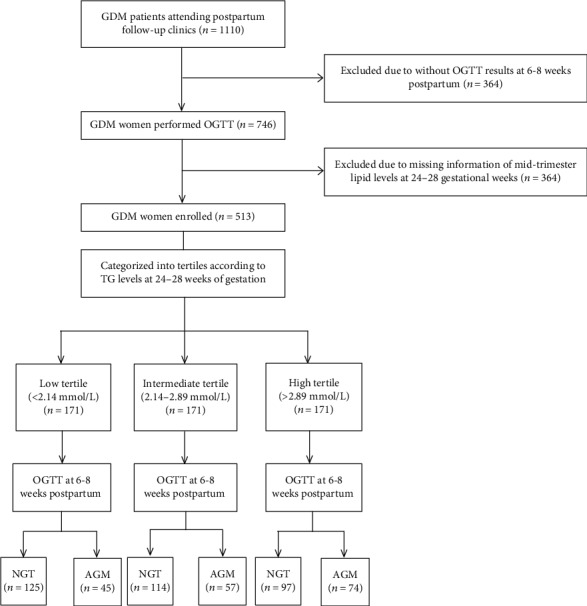
Subject screening and distribution. GDM: gestational diabetes mellitus; OGTT: oral glucose tolerance test; TG: triglyceride; NGT: normal glucose tolerance; AGM: abnormal glucose metabolism.

This was a retrospective cohort study with 1110 GDM patients attending the postpartum follow-up clinics in Shanghai General Hospital between April 2012 and April 2019. In the present analysis, we excluded 364 women who did not attend the OGTT at 6-8 weeks postpartum and 233 women who did not test midtrimester lipid levels. The remaining 513 GDM women were included in the final analysis (Figure 1). Each enrolled pregnant woman was administered a 75 g OGTT at 24–28 weeks of gestation and 6–12 weeks after delivery. In addition, fasting lipid levels, including serum total cholesterol (TC), TGs, high-density lipoprotein-cholesterol (HDL-C), and low-density lipoprotein-cholesterol (LDL-C), insulin levels, alanine aminotransferase (ALT), and aspartate aminotransferase (AST) were detected. Women were diagnosed with GDM if one or more plasma glucose values during the 75 g OGTT at 24–28 gestational weeks were met or exceeded: 0 h, 5.1 mmol/L; 1 h, 10.0 mmol/L; and 2 h, 8.5 mmol/L [14]. The subjects underwent a 75 g OGTT at 6–8 weeks after delivery, and the diagnosis of normal glucose tolerance (NGT), T2DM, impaired fasting glucose (IFG), and impaired glucose tolerance (IGT) was based on the World Health Organization (WHO) diagnostic criteria published in 1999 [15]. According to TG levels after delivery, the patients were diagnosed with normal TG (NTG, TG < 1.70 mmol/L) and abnormal TG (ATG, TG ≥ 1.70 mmol/L) [16]. Each patient provided informed consent, and the research was carried out in compliance with the Declaration of Helsinki. The study protocol was approved by the ethics committee of Shanghai General Hospital, Shanghai Jiao Tong University.

#### 2.1.1. Grouping

(1) All GDM patients were categorized into tertiles according to their TG levels at 24–28 weeks of gestation as follows: low tertile (<2.14 mmol/L), intermediate tertile (2.14–2.89 mmol/L), and high tertile (>2.89 mmol/L). (2) The patients were also categorized into two groups according to the results of the postpartum OGTT: the NGT group (fasting plasma glucose (FPG) < 6.1 mmol/L and 2 h glucose load plasma glucose (2hPG) < 7.8 mmol/L) and the AGM group (FPG ≥ 6.1 mmol/L or 2hPG ≥ 7.8 mmol/L) (Figure 1).

### 2.2. Data Collection

The following maternal characteristics were assessed: age at delivery, height, body weight before pregnancy and at 6–8 weeks postpartum, first-degree family history of diabetes, and insulin treatment during pregnancy.

### 2.3. Metabolic Measurements

Plasma glucose levels were measured enzymatically. Fasting insulin (FINS) and serum lipid (TC, TG, HDL-C, and LDL-C) levels were determined by chemiluminescent assays. ALT and AST levels were determined by enzymatic methods. HbA1c and glycated albumin (GA) levels were determined by high-performance liquid chromatography. HOMA-*β* and HOMA-IR were calculated to evaluate *β* cell function and insulin resistance using the following formulas: HOMA‐*β* = 20 × FINS (*μ*U/mL)/(FPG (mmol/L) − 3.5) and HOMA‐IR = FPG (mmol/L) × FINS (*μ*U/mL)/22.5 [17].

### 2.4. Statistical Analysis

Data were expressed as the mean ± standard deviation for normally distributed variables and as the median with the interquartile range for skewed data. To determine the differences among the three groups, we conducted analysis of variance or the Kruskal–Wallis test for continuous variables and a chi-square test for categorical variables. Least significance difference tests were used to perform pairwise comparisons between two groups. Logistic regression analysis was used to determine whether elevated TG levels during gestation were independently associated with AGM. Statistical significance was set at *p* < 0.05. Statistical analyses were carried out using SPSS (IBM Corp., Armonk, NY).

## 3. Results

### 3.1. Demographic and Metabolic Characteristics of GDM Patients at 24-28 Gestational Weeks, Categorized into Tertiles according to TG Levels at the Time of Measurement

There were significant differences among the three groups concerning metabolic levels during pregnancy. The participants in the high TG tertile had a higher prepregnancy BMI (23.74 ± 3.47 kg/m^2^ versus 22.12 ± 3.54 kg/m^2^, *p* < 0.001), higher FPG levels (5.25 ± 0.97 mmol/L versus 4.82 ± 0.80 mmol/L, *p* < 0.001), higher 1 h plasma glucose (1hPG) levels (10.58 ± 1.94 mmol/L versus 9.73 ± 1.86 mmol/L, *p* < 0.001), higher 2 h plasma glucose (2hPG) levels (9.13 ± 2.03 mmol/L versus 8.29 ± 1.81 mmol/L, *p* < 0.001), higher HbA1c (5.47 ± 0.58% versus 5.28 ± 0.49%, *p* = 0.006), higher TC levels (5.85 ± 1.23 mmol/L versus 5.15 ± 0.97 mmol/L, *p* = 0.026), lower HDL-C levels (1.64 ± 0.34 mmol/L versus 1.88 ± 0.47 mmol/L, *p* < 0.001) and higher HOMA-IR (2.36 (1.62-3.45) versus 1.49 (0.97-2.33), *p* < 0.001) than the participants in the low TG tertile (Table 1).

### 3.2. Glucose and Lipid Levels of GDM Patients at 6–8 Weeks Postpartum

After delivery, approximately 34.5% (177/513) of the GDM patients had sustained high glucose levels. The rates of IFG, IGT, IFG combined with IGT, and DM were 1.2% (6/513), 24.0% (123/513), 2.7% (14/513), and 6.6% (34/513), respectively. The TG levels of most GDM patients returned to normal 6–8 weeks after delivery; however, 25.0% (128/513) of the women continued to have hypertriglyceridemia.

### 3.3. Demographic and Metabolic Characteristics of GDM Patients at 6-8 Weeks Postpartum Categorized into Tertiles according to TG Levels at 24-28 Gestational Weeks

There were significant differences among the three groups concerning metabolic levels after delivery. The participants in the high TG tertile showed a higher postpartum BMI (25.03 ± 3.14 kg/m^2^ versus 23.56 ± 3.30 kg/m^2^, *p* < 0.001), higher FPG levels (5.18 ± 0.90 mmol/L versus 4.87 ± 0.68 mmol/L, *p* = 0.002), higher 1hPG levels (9.66 ± 2.43 mmol/L versus 9.11 ± 2.15 mmol/L, *p* = 0.048), higher 2hPG levels (7.64 ± 2.36 mmol/L versus 6.99 ± 1.98 mmol/L, *p* = 0.022), higher TC levels (5.36 ± 0.91 mmol/L versus 5.09 ± 0.99 mmol/L, *p* = 0.026), higher LDL-C levels (3.39 ± 0.73 mmol/L versus 3.04 ± 0.92 mmol/L, *p* = 0.001), lower HDL-C levels (1.34 ± 0.85 mmol/L versus 1.54 ± 0.32 mmol/L, *p* < 0.001) and higher HOMA-IR (1.59 (0.97-2.43) versus 1.10 (0.69-1.63), *p* < 0.001) than the participants in the low TG tertile. In addition, the prevalence rates of AGM were 43.27%, 33.33%, and 26.90% (*p* = 0.005) in the three groups (TG > 2.89 mmol/L, TG: 2.14–2.89 mmol/L, and TG < 2.14 mmol/L), respectively (Table 2).

### 3.4. Elevated TG Levels as a Biomarker for the Development of Postpartum AGM in GDM Patients

To determine whether the elevated TG level during the second trimester of pregnancy was a biomarker for postpartum AGM, the odds ratios (ORs) for AGM in women with different TG levels during the second trimester were calculated. First, compared to the low TG tertile group at 24–28 gestational weeks, the crude OR for AGM was 2.073 (95% CI 1.317–3.263, *p* = 0.002) for the high TG tertile group, and the adjusted OR was 2.001 (95% CI 1.054–3.800, *p* = 0.034) when considering age, prepregnancy BMI, family DM history, insulin treatment during pregnancy, FPG during the second trimester, HbA1c during the second trimester, macrosomia, ALT, and AST (Table 3). In addition to elevated TG levels, we also found that HbA1c during pregnancy (OR 2.738, 95% CI 1.415–5.299, *p* = 0.003) was a risk factor for postpartum AGM. However, no association was found between other factors and postpartum AGM.

**Figure 2:**
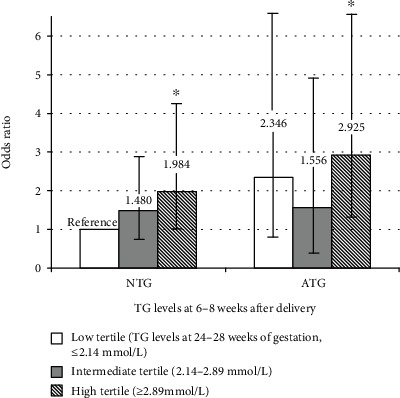
Odds ratios and 95% confidence intervals for postpartum AGM associated with TG levels (the interaction during pregnancy and the postpartum period). AGM: abnormal glucose metabolism; TG: triglyceride; NTG: normal TG levels after delivery (<1.70 mmol/L); ATG: abnormal TG levels after delivery (≥1.70 mmol/L). ^∗^Compared to the reference group, *p* < 0.05.

Then, the TG levels after delivery were also determined. We examined the interaction term between TG levels during pregnancy and the postpartum period, and the result was statistically significant (*p* < 0.05). Therefore, we examined the association between TG levels during the second trimester and the risk of postpartum AGM stratified by TG levels after delivery. Women both in the high TG tertile during pregnancy and with ATG levels postpartum were three times as likely as those in the low tertile with NTG levels postpartum (OR 2.925, 95% CI 1.271–6.730, *p* = 0.012) to develop AGM postpartum. Moreover, women in the high tertile with NTG levels postpartum were twice as likely as those in the low tertile with NTG levels postpartum (OR 1.984, 95% CI 1.022–4.365, *p* = 0.046) to develop AGM postpartum (Figure 2). Therefore, the association with postpartum AGM was strong among women with high TG levels in the second trimester, regardless of whatever TG levels after delivery.

## 4. Discussion

In this study, we found that GDM patients with elevated TG levels during pregnancy not only had more severe glucose and lipid disorders and insulin resistance at the time of the measurement but also were at an increased risk for the development of postpartum AGM.

The incidence of AGM after GDM had been reported to vary from 2.6% to 38% within 12 weeks after delivery, which was significantly higher than NGT women during pregnancy [18–20]. Another study showed that approximately 70% of GDM patients would eventually develop DM [21]. Therefore, it is important to determine the risk factors for progression to T2DM over the long term [22]. A systematic review showed that prepregnancy BMI, family history of diabetes, advanced maternal age, increased HbA1c levels, and increased insulin use during pregnancy were associated with the future development of T2DM in GDM patients [23]. During pregnancy, some studies found that high TG levels during gestation were independently associated with an increased risk of GDM [24, 25]. Furthermore, Kim et al. suggested that postpartum TG was one risk factor for postpartum glucose intolerance [26]. We evaluated the TG levels both during midterm and after delivery to show that TG levels during pregnancy might be an independent biomarker for long-term glucose metabolism in GDM patients, which might be more meaningful compared to TG levels after delivery. Hypertriglyceridemia during pregnancy could not only induce acute pancreatitis at the time [27] but also was associated with increased risk of pregnancy-induced hypertension and preterm labor [28, 29]. The midterm elevated TG levels and association with GDM glucose outcome after delivery reminded us to pay more attention to hypertriglyceridemia during pregnancy in GDM patients to prevent postpartum DM.

The serum levels of lipids, including TC, TG, LDL-C, HDL-C, and phospholipids, gradually increase starting in the 12th week of pregnancy and show more pronounced increases during the second and third trimesters [30–32]. Exaggerated TG rises have been found in GDM patients in all trimesters of pregnancy compared to women with NGT [33]. We found that GDM patients with elevated TG levels in the midterm of pregnancy had higher glucose levels and more severe insulin resistance during both pregnancy and the postpartum period. Free fatty acids (FFAs) potentially derived from elevated TGs might decrease insulin sensitivity, creating a vicious cycle between TG levels and insulin resistance [8, 34, 35]. Excess FFAs could result in the generation of toxic lipids, including diacylglycerides and ceramides. These toxic lipids contribute to endoplasmic reticulum stress, mitochondrial dysfunction, and the generation of reactive oxygen species, which together trigger inflammation and insulin resistance [36–39]. FFAs also impact the fatty acid composition of cellular membranes. This directly affects cell function as well as the incorporation of insulin receptors into the membrane [40, 41]. A higher concentration of TGs during pregnancy leads to subsequent impairment of glucose regulation mainly via FFAs, but further research is needed to explore the detailed mechanism.

This study had some strengths and limitations. This was a study focusing on the association between TG levels and the risk of AGM after delivery, which was a novel perspective. The TG levels were measured at two time points, namely, during pregnancy and the postpartum period, revealing that TG levels during pregnancy were independent factors that affect postpartum glucose metabolism. However, we did not collect information about the lifestyles of our participants, such as dietary factors and physical activity, which could act as confounders. In addition, we did not measure FFA levels, which might be relevant to detailed mechanisms because this measurement was not included in the routine checklist in the clinic. It would be preferable if the TG levels during early and late pregnancy were measured, which would help us to assess the changes in TG levels during different trimesters and the influence of these changes on postpartum glucose metabolism.

In conclusion, our data showed that elevated TG levels during the second trimester were associated with an increased risk of postpartum AGM among women with previous GDM. For those patients, a low-fat diet, lifestyle modifications, and intensified follow-up are strongly recommended to prevent postpartum DM.

## Figures and Tables

**Table 1:** Demographic and metabolic characteristics of GDM patients at 24-28 gestational weeks, categorized into tertiles according to TG levels at the time of measurement.

Variables	Low tertile (<2.14 mmol/L) (*N* = 171)	Intermediate tertile (2.14~2.89 mmol/L) (*N* = 171)	High tertile (>2.89 mmol/L) (*N* = 171)	*p* value
Prepregnancy BMI (kg/m^2^)	22.12 ± 3.54	22.78 ± 3.21^∗^	23.74±3.47^∗∗^	0.0001
FPG (mmol/L)	4.82 ± 0.80	4.90 ± 0.79	5.25±0.97^∗∗^	0.0001
1hPG (mmol/L)	9.73 ± 1.86	10.23 ± 1.67	10.58±1.94^∗∗^	0.0001
2hPG (mmol/L)	8.29 ± 1.81	8.68±1.84^∗∗^	9.13±2.03^∗∗^	0.0001
HbA1c (%)	5.28 ± 0.49	5.33 ± 0.48	5.47±0.58^∗∗^	0.006
GA (%)	13.60 ± 1.76	13.46 ± 2.00	13.28 ± 2.09	0.396
TC (mmol/L)	5.15 ± 0.97	5.75 ± 1.04	5.85 ± 1.23^∗^	0.016
HDL-C (mmol/L)	1.88 ± 0.47	1.80 ± 0.34	1.64±0.34^∗∗^	0.0001
LDL-C (mmol/L)	3.12 ± 0.72	3.25 ± 0.80	3.17 ± 0.98	0.356
ALT (U/L)	12.20 (9.43-18.08)	12.15 (9.30-19.75)	10.90 (9.05-16.60)	0.574
AST (U/L)	16.10 (13.23-19.78)	16.05 (13.60-20.78)	15.40 (12.50-19.90)	0.483
HOMA-*β* (%)	123.87 (87.15-167.03)	139.73 (91.90-198.51)	124.10 (91.10-179.72)	0.290
HOMA-IR	1.49 (0.97-2.33)	1.89 (1.31-2.75)^∗∗^	2.36 (1.62-3.45)^∗∗^	0.0001

Data are expressed as the mean ± SD or median (interquartile range). TG: triglyceride; BMI: body mass index; FPG: fasting plasma glucose; 1hPG: 1 h plasma glucose; 2hPG: 2 h plasma glucose; HbA1c: hemoglobin A1c; GA: glycated albumin; TC: total cholesterol; HDL-C: high-density lipoprotein-cholesterol; LDL-C: low-density lipoprotein-cholesterol; ALT: alanine aminotransferase; AST: aspartate aminotransferase; HOMA-IR: homeostasis model assessment for insulin resistance index; HOMA-*β*: homeostasis model assessment for *β* cell function. ^∗^Compared to the low tertile group, *p* < 0.05. ^∗∗^Compared to the low tertile group, *p* < 0.01.

**Table 2:** Demographic and metabolic characteristics of GDM patients at 6-8 weeks postpartum categorized into tertiles according to TG levels at 24-28 gestational weeks.

Variables	Low tertile (<2.14 mmol/L) (*N* = 171)	Intermediate tertile (2.14~2.89 mmol/L) (*N* = 171)	High tertile (>2.89 mmol/L) (*N* = 171)	*p* value
Age (years)	31.49 ± 4.45	32.07 ± 4.90	32.29 ± 4.07	0.140
Postpartum BMI (kg/m^2^)	23.56 ± 3.30	24.018 ± 3.37	25.03±3.14^∗∗^	0.0001
FPG (mmol/L)	4.87 ± 0.68	4.97 ± 0.70	5.18±0.90^∗∗^	0.001
1hPG (mmol/L)	9.11 ± 2.15	9.19 ± 2.15	9.66 ± 2.43^∗^	0.048
2hPG (mmol/L)	6.99 ± 1.98	7.29 ± 2.12	7.64±2.36^∗∗^	0.022
HbA1c (%)	5.55 ± 0.46	5.52 ± 0.45	5.62 ± 0.44	0.228
GA (%)	13.63 ± 1.38	13.21 ± 1.24	13.36 ± 1.26	0.090
TC (mmol/L)	5.09 ± 0.99	5.14 ± 0.97	5.36 ± 0.91^∗^	0.026
TG (mmol/L)	1.05 ± 0.57	1.23 ± 0.82^∗^	1.72±0.97^∗∗^	0.0001
HDL-C (mmol/L)	1.54 ± 0.32	1.53 ± 0.39	1.34±0.85^∗∗^	0.0001
LDL-C (mmol/L)	3.04 ± 0.92	3.08 ± 0.78	3.39±0.73^∗∗^	0.001
HOMA-*β* (%)	84.51 (55.46-131.82)	88.50 (54.81-142.38)	90.95 (59.12-154.91)	0.120
HOMA-IR	1.10 (0.69-1.63)	1.21 (0.83-1.93)	1.59 (0.97-2.43)^∗∗^	0.0001
DM family history (%)	54 (31.58)	44 (25.73)	53 (30.99)	0.426
Insulin treatment therapy (%)	48 (28.07)	58 (33.92)	65 (38.01)	0.147
Postpartum AGM (%)	46 (26.90)	57 (33.33)	74 (43.27)^∗∗^	0.006
Macrosomia (%)	9 (5.26)	16 (9.36)	9 (5.26)	0.213

Data are expressed as the mean ± SD or median (interquartile range). TG: triglyceride; BMI: body mass index; FPG: fasting plasma glucose; 1hPG: 1 h plasma glucose; 2hPG: 2 h plasma glucose; HbA1c: hemoglobin A1c; GA: glycated albumin; TC: total cholesterol; HDL-C: high-density lipoprotein-cholesterol; LDL-C: low-density lipoprotein-cholesterol; DM: diabetes mellitus; AGM: abnormal glucose metabolism; HOMA-IR: homeostasis model assessment for insulin resistance index; HOMA-*β*: homeostasis model assessment for *β* cell function. ^∗^Compared to the low tertile group, *p* < 0.05. ^∗∗^Compared to the low tertile group, *p* < 0.01.

**Table 3:** Elevated TG levels as a biomarker for the development of postpartum AGM in GDM patients.

Variables	OR	95% CI	*p* value
TG levels at 24-28 gestational weeks (mmol/L)			
Low tertile	*Reference*		
Intermediate tertile	1.120	0.597-2.100	0.724
High tertile	**2.001**	**1.054-3.800**	**0.034**
Age (years)	1.046	0.986-1.110	0.135
Prepregnancy BMI (kg/m^2^)	0.980	0.896-1.072	0.661
Family history of DM	1.007	0.550-1.841	0.983
Insulin treatment during pregnancy	1.460	0.816-2.611	0.202
FPG at 24-28 gestational weeks (mmol/L)	0.852	0.570-1.275	0.437
HbA1c at 24-28 gestational weeks (%)	**2.738**	**1.415-5.299**	**0.003**
Macrosomia	1.011	0.380-2.691	0.983
ALT at 24-28 gestational weeks (U/L)	0.985	0.953-1.018	0.371
AST at 24-28 gestational weeks (U/L)	1.019	0.978-1.061	0.368

TG: triglyceride; DM: diabetes mellitus; BMI: body mass index; FPG: fasting plasma glucose; HbA1c: hemoglobin A1c; ALT: alanine aminotransferase; AST: aspartate aminotransferase.

## Data Availability

The data used to support the findings of this study are available from the corresponding author upon request.
